# MUSET: set of utilities for constructing abundance unitig matrices from sequencing data

**DOI:** 10.1093/bioinformatics/btaf054

**Published:** 2025-02-03

**Authors:** Riccardo Vicedomini, Francesco Andreace, Yoann Dufresne, Rayan Chikhi, Camila Duitama González

**Affiliations:** GenScale, Université de Rennes, Inria RBA, CNRS UMR 6074, F-35000 Rennes, France; Institut Pasteur, Université Paris Cité, Sequence Bioinformatics Unit, F-75015 Paris, France; Sorbonne Université, Collège Doctoral, F-75005 Paris, France; Institut Pasteur, Université Paris Cité, Sequence Bioinformatics Unit, F-75015 Paris, France; Sorbonne Université, Collège Doctoral, F-75005 Paris, France; Institut Pasteur, Université Paris Cité, Sequence Bioinformatics Unit, F-75015 Paris, France; Institut Pasteur, Université Paris Cité, Sequence Bioinformatics Unit, F-75015 Paris, France

## Abstract

**Summary:**

MUSET is a novel set of utilities designed to efficiently construct abundance unitig matrices from sequencing data. Unitig matrices extend the concept of k-mer matrices by merging overlapping k-mers that unambiguously belong to the same sequence. MUSET addresses the limitations of current software by integrating k-mer counting and unitig extraction to generate unitig matrices containing abundance values, as opposed to only presence–absence in previous tools. These matrices preserve variations between samples while reducing disk space and the number of rows compared to k-mer matrices. We evaluated MUSET’s performance using datasets derived from a 618-GB collection of ancient oral sequencing samples, producing a filtered unitig matrix that records abundances in <10 h and 20 GB memory.

**Availability and implementation:**

MUSET is open source and publicly available under the AGPL-3.0 licence in GitHub at https://github.com/CamilaDuitama/muset. Source code is implemented in C++ and provided with kmat_tools, a collection of tools for processing k-mer matrices. Version v0.5.1 is available on Zenodo with DOI 10.5281/zenodo.14164801.

## 1 Introduction

Unitigs are biological sequences that compactly and exhaustively represent sequencing data or assembled genomes. They are constructed from k-mers, but unlike k-mers, they avoid the redundancy problem of multiple overlapping sequences covering the same genomic locus. They have proven useful for analyzing genomic diversity across several sequencing datasets (Iqbal *et al.* 2012, Jaillard *et al.* 2018, Cracco and Tomescu 2023). A more formal definition characterizes unitigs as maximal nonbranching paths in a de Bruijn graph (dBG). The de Bruijn graph is a fundamental data structure in bioinformatics and is widely used in many genomics applications (Compeau *et al.* 2011, Lopez-Maestre *et al.* 2016, Andreace *et al.* 2023). It represents a set of distinct k-mers (substrings of size k) and their k−1 prefix-suffix overlaps as a graph (Chikhi *et al.* 2022). The process of generating maximal unitigs from k-mers, known as *compaction*, consists of consolidating all maximal nonbranching paths of k-mers into single strings (Chikhi *et al.* 2022, Marchet 2022). The resulting graph is called compacted dBG. In this work, the term “unitigs” will always refer to the deterministically unique set of maximal unitigs.

A unitig matrix is a data structure representing sequence content across multiple experiments by recording a numerical value for each unitig across all samples. In a scenario involving a collection S of samples, a binary unitig matrix M has elements M(i,j) that indicate the presence (1) or absence (0) of unitig i in sample j. In other words, rows are unitigs, and columns are samples.

### 1.1 Related work

Numerous cutting-edge tools that rely on the construction of a de Bruijn graph for unitig computation have been developed over the years, including BCALM (Chikhi *et al.* 2016), Cuttlefish (Khan *et al.* 2022), Bifrost (Holley and Melsted 2020), and GGCAT (Cracco and Tomescu 2023). By computing the unitigs of a union of multiple samples, one can construct a binary unitig matrix. However, current software overlooks the concept of abundance, which estimates the frequency with which a unitig appears in a sample.

BCALM and Cuttlefish simply produce a set of unitigs, i.e. a *compacted* dBG, without recording the sample of origin. GGCAT and Bifrost, on the other hand, can build *colored compacted* dBGs, a variant of dBGs that additionally keeps track of the source of each k-mer (Iqbal *et al.* 2012). Such graphs are implicitly binary unitig matrices. Notably, GGCAT has demonstrated superior performance to its counterparts (Cracco and Tomescu 2023).

Unitig matrices are data structures analogous to k-mer matrices, which are commonly used to represent sequence content across multiple experiments, e.g. for indexing environmental metagenomes (Lemane *et al.* 2024), source attribution of pathogenic bacteria via supervised learning (Castelli *et al.* 2023), transcriptomic analyses (Silva *et al.* 2023), contamination removal, and contamination assessment of ancient metagenomic data (Duitama González *et al.* 2023, González *et al.* 2023). While k-mer matrices can be constructed efficiently [e.g. using kmtricks (Lemane *et al.* 2022)], storing and processing large k-mer matrices remains challenging, especially when integrating them with existing libraries for machine learning, dimensionality reduction, or visualization. As an alternative, unitig matrices offer a more compact and manageable representation, preserving the variations between samples while optimizing disk space usage and processing time. Individual k-mer counts can be naturally averaged along a unitig to produce a single abundance value per unitig, robustly approximating the counts of all k-mers from that unitig (Marchet et al. 2020).

Using unitigs instead of k-mers can reduce multiple testing burden, a common challenge in the study of genomic variation across datasets (Jaillard *et al.* 2018). Presence–absence unitig matrices have proven useful for statistical analyses in human genomic studies, where the frequency of sequences that characterize certain species can effectively distinguish between phenotypes more accurately than their mere presence or absence (Frouin *et al.* 2023). Unitig matrices also have the potential to be useful for RNA-Seq differential expression analyses in transcriptomics, where low-coverage unitigs can help identify sequencing errors. However, to our knowledge, no software effectively addresses the gap that exists in the construction of abundance unitig matrices, as opposed to presence–absence unitig matrices. For this reason, we introduce MUSET, a pipeline designed for the practical construction of abundance unitig matrices. Along with it, we additionally provide kmat_tools, a comprehensive suite of tools (internally exploited by MUSET) for manipulating k-mer matrices, and muset_pa, a pipeline that uses GGCAT and kmat_tools to rapidly build a presence–absence unitig matrix with no k-mer filtering.

## 2 Tool description and evaluation

### 2.1 MUSET overview

**Figure 1.**
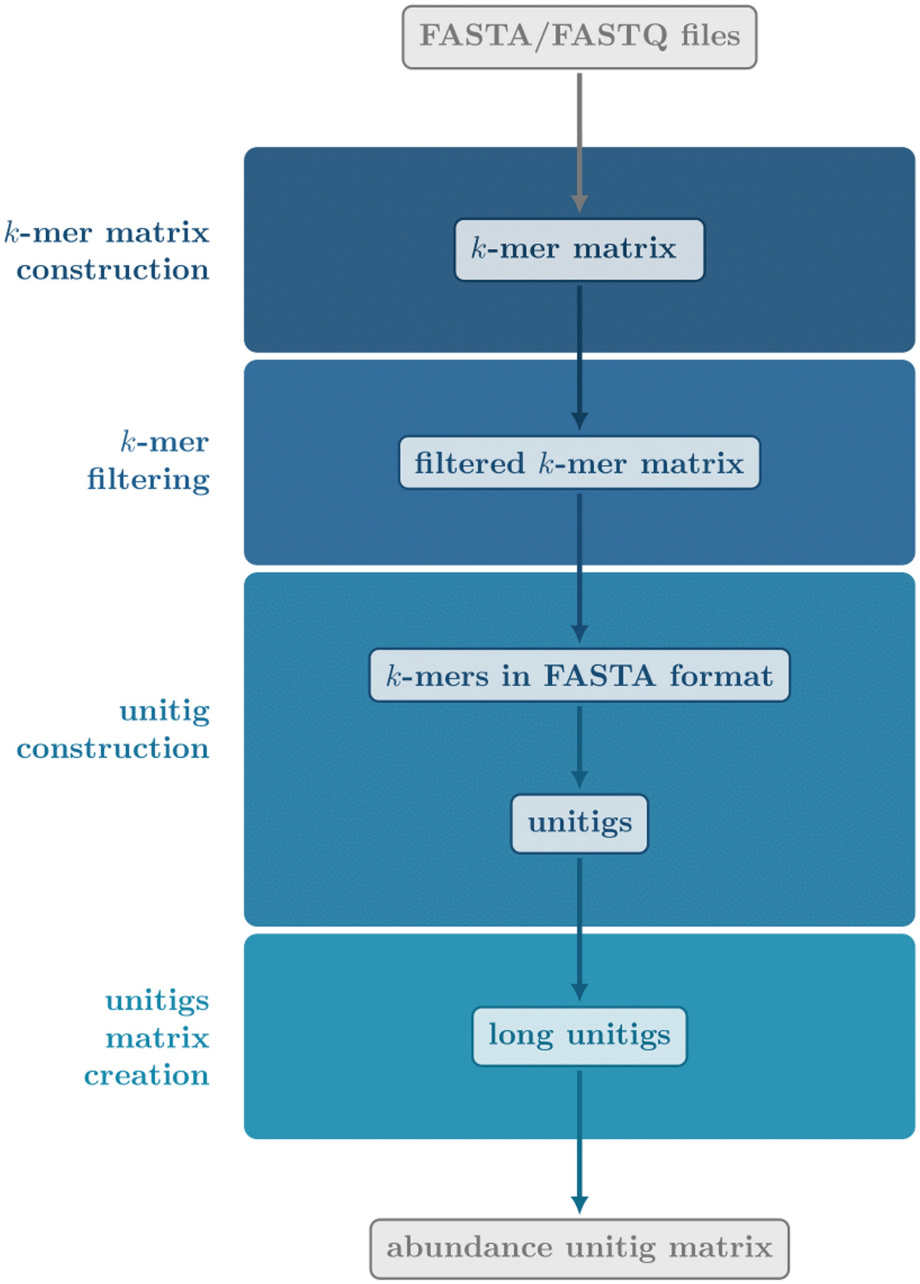
MUSET pipeline. It consists of four main steps: *k*-mer matrix construction, *k*-mer matrix filtering, unitig creation, and unitig matrix creation.

MUSET leverages kmtricks (Lemane *et al.* 2022) for efficient k-mer counting over large collections of genomic sequences provided as FASTA/FASTQ files. It then uses GGCAT (Cracco and Tomescu 2023) for unitig construction and SSHash (Pibiri 2022) to assign k-mer counts to unitigs. Intermediate filtering steps are performed at both k-mer and unitig levels. The final output is a unitig matrix where rows correspond to unitigs, columns correspond to samples, and each element encodes the average abundance and fraction of the unitig’s k-mers present in a sample. The pipeline is depicted in Fig. 1. It consists of the following main steps explained below: (i) k-mer matrix construction, (ii) k-mer matrix filtering, (iii) unitig construction, and (iv) unitig matrix construction. All the commands shown below are automated by MUSET.

#### 2.1.1 k-mer matrix construction

kmtrick’s pipeline is run to build a k-mer matrix from the input FASTA/Q files. The matrix is stored in partitions using a custom lz4-compressed binary format. Although not used in the elaboration of this paper, we recently added the possibility to input unitigs from the Logan project (Chikhi *et al.* 2024) instead of FASTA/FASTQ files.

#### 2.1.2 k-mer matrix filtering

kmtricks’s matrix partitions are filtered concurrently and merged into a single k-mer matrix in text format. The filtering aims to retain k-mers that potentially reflect differences between samples. More precisely, by default, we keep k-mers present in at least 10% of the samples and absent in at least 10% of the samples. Users can set custom values to both thresholds by providing a fraction or an absolute number.

#### 2.1.3 Unitig construction

The k-mers of the filtered matrix are outputted in FASTA format (command kmat_tools fasta) to build a set of unitigs with GGCAT. Unitigs shorter than a certain threshold (2k−1 base pairs by default) are later discarded using the command kmat_tools fafmt.

#### 2.1.4 Unitig matrix construction

This step involves executing the command kmat_tools unitig to create an abundance unitig matrix. First, an SSHash-based dictionary is built from the (filtered) unitigs to assign each k-mer to the unitig it belongs to. The filtered k-mer matrix is then processed to extract abundance values and k-mer presence while simultaneously aggregating k-mer data at the unitig level. The aggregation is done for each unitig by computing the fraction of the unitig’s k-mers belonging to a given sample and their average abundance.

More precisely, the fraction of k-mers in a unitig u that are present in a sample S is defined as:

(1) $$f(u,S)=\frac{\sum_{i=1}^{N}x_{i}}{N}$$

where N is the number of k-mers in u, and $x_{i}$ is a binary variable, i.e. 1 when the *i*th k-mer is present in sample S and 0 otherwise.

The average abundance of a unitig u with respect to a sample S is defined as:

(2) $$A(u,S)=\frac{\sum_{i=1}^{N}c_{i}}{N}$$

where N is the number of k-mers in u, and $c_{i}$ is the abundance of the i-th k-mer of u in sample S.

### 2.2 Evaluation

We evaluated the performance of MUSET (Table 1) on two datasets composed of a collection of 360 ancient oral samples and their potential contaminants (Duitama González *et al.* 2023) using a default k value of 31. This collection is made of compressed FASTQ files, which add up to 618 GB. The first dataset, referred to as the small dataset, consists of a subset of rows of the full k-mer matrix obtained from the aforementioned collection. The small-dataset matrix contains ≈14.3 million k-mers and has a size of 11 GB. The second dataset, denoted as the large dataset, encompasses the entire matrix. The large-dataset matrix contains ≈64.6 billion k-mers and has a size of 1.4 TB. The instructions to reproduce our results are available at the following link: https://github.com/CamilaDuitama/muset/tree/main/reproducibility.

**Table 1.** Running time, peak memory, and disk usage of MUSET on the small and large datasets.^a^

	Small dataset (11 GB)			Large dataset (618 GB)		
MUSET step	Wall-clock time	Peak memory (GB)	Disk usage (GB)	Wall-clock time	Peak memory (GB)	Disk usage (GB)
*k*-mer matrix construction	3 h 6 min 46 s	2.4	11	7 h 47 min 59 s	19	1437
*k*-mer filtering	0 h 2 min 04 s	< 0.1	0.1	1 h 37 min 25 s	< 0.1	80
Unitig creation (GGCAT)	0 h 0 min 28 s	0.7	< 0.1	0 h 2 min 28 s	1.2	0.9
Unitig matrix creation	0 h 0 min 01 s	< 0.1	< 0.1	0 h 15 min 20 s	2.3	2.7

Total	3 h 8 min 10 s	2.4	11.2	9 h 43 min 12 s	19	1525

a The two datasets are derived from the same collection of FASTQ files of ancient DNA reads. The small dataset corresponds to a subset of the *k*-mer matrix of the large dataset. Wall-clock time and memory usage were computed using 20 threads.

To our knowledge, no other tool can directly create an abundance unitig matrix from sequencing data. The most similar state-of-the-art tool to produce analogous presence–absence matrices is GGCAT (which we happen to use inside MUSET). For this reason, we compared MUSET to a stand-alone execution of GGCAT by running both tools on the large test dataset.

In Table 2, MUSET demonstrates superior performance over GGCAT in processing the large metagenomic dataset, creating a filtered abundance unitig matrix more than twice as fast (9.7 h versus 24.3 h), using 88% less memory (19 GB versus 167 GB), yet at the expense of a higher disk usage (1.5 TB versus 641 GB). Notably, MUSET can reduce the size of the k-mer matrix by 2 (small dataset) to 3 (large dataset) orders of magnitude when converting it to a unitig matrix. MUSET not only achieves shorter running times and lower peak memory usage than GGCAT due to its upstream k-mer filter, but it is also more convenient and informative as it directly produces an abundance unitig matrix. GGCAT instead produces a colored compacted dBG in text format with colors in binary format, and the user would have to generate a presence–absence unitig matrix on their own: this is now directly achievable using the muset_pa tool we provide.

**Table 2.** Comparison of running time, peak memory, and disk usage between MUSET (filtered unitig matrix) and GGCAT (unfiltered unitigs) on the large dataset.^a^

Method	Wall-clock time	Peak memory	Disk usage
**MUSET**	**9 h 43 min 12 s**	**19 GB**	**1.5 TB**
GGCAT	24 h 20 min 40 s	167 GB	641 GB

a The large dataset consists of a 618-GB collection of 360 samples (compressed FASTQ files) from an ancient metagenomic dataset. Tools were run using 20 threads. Results for MUSET are provided in bold.

## 3 Discussion and conclusions

Abundance unitig matrices provide a compact, manageable, and more informative representation of sequencing data across multiple samples, offering a valuable resource for downstream analyses in large-scale (meta/pan)genomic studies. Besides, the representation of sequencing data as abundance unitig matrices offers a versatile option with potential applications in various contexts, from sequencing error filtering and indexing to reference-free comparison of sequencing samples. It also provides a useful input for supervised and unsupervised learning models built on large-scale sequencing datasets.

The most time-consuming and memory-intensive step in MUSET is the k-mer matrix construction, which can be optionally skipped if users have already created their own k-mer matrix in text format. This is followed by the k-mer filtering step (see Table 1). While k-mer filtering can theoretically be bypassed by setting appropriate parameters, doing so will increase disk space and peak memory usage in subsequent pipeline steps. We recommend applying this k-mer filtering to discard “uninformative” k-mers and reduce the computational burden. However, stringent filtering may interfere with the biological interpretation of unitigs by possibly yielding chimeric sequences, which is worth investigating and mitigating in future work.

To evaluate if the fraction of k-mers of a unitig present in a sample [see Equation (1)] varied with respect to unitig length, we plotted this fraction against various unitig lengths estimated on the small dataset. When considering k-mer frequencies larger or equal than 0.1, per-sample mean fraction of present k-mers in a unitig remains roughly around 80% regardless of unitig length, as shown in Supplementary Fig. S1. For this reason, we believe there is little variation in unitig abundance estimation with respect to unitig length; in other words, roughly 80% of the k-mers in a sample are always present, independently of unitig length. Additionally, we evaluated the possible changes introduced by increasing variation in the input sequences by experimenting with 10, 100, 1000, 2000, and 3000 *E. coli* genomes from AllTheBacteria (Hunt *et al.* 2024). As seen in Supplementary Table S1, with an increasing number of assembled genomes, hence an increasing number of SNPs, both the number of k-mers and unitigs proportionally grow, with the unitig/k-mer ratio remaining relatively consistent across different sample sizes. Average unitig length, as expected, decreases since insertions or deletions in the input sequences should split the initial unitigs into smaller fragments.

It is important to point out two clarifications for the reader. First, MUSET currently constructs a union dBG and cannot update the matrix by adding a new sample. Second, our method’s improvement in computation time comes with the initial filtering of k-mers. Both points might be helpful for future research.

To conclude, MUSET addresses the technical gap associated with generating abundance unitig matrices to represent extensive collections of sequencing datasets. The tool utilizes kmtricks for efficient indexing and k-mer counting, GGCAT for constructing unitigs, and SSHash to efficiently map k-mers to unitigs. Ultimately, MUSET reports the abundance of each unitig in the collection of samples by estimating the fraction of observed k-mers relative to the total number of k-mers that constitute each unitig, in addition to calculating the average abundance per sample [see Equation (2)]. We anticipate that MUSET will be a valuable replacement to k-mer matrices of sequencing data. By providing a tool that offers a smaller representation with equivalent information content, MUSET has the potential to accelerate and facilitate the provision of biologically significant and reference-free insights into sequencing data.

## Supplementary Material

btaf054_Supplementary_Data

## Data Availability

MUSET is open source and implemented in C++. It is provided with kmat_tools, a collection of tools for processing k-mer matrices. MUSET v0.5.1 is publicly available on Zenodo with DOI 10.5281/zenodo.14164801. The latest available version with the instructions for installing and running it are available at https://github.com/CamilaDuitama/muset.
