# Supplementary material for: MUSET: set of utilities for constructing abundance unitig matrices from sequencing data
Source: Bioinformatics. 2025 Feb 3;41(3):btaf054. doi: 10.1093/bioinformatics/btaf054 (PMC11897428; doi:10.1093/bioinformatics/btaf054)
Supplement: btaf054_Supplementary_Data [file btaf054_supplementary_data.zip › Supplementary_file_to_muset.pdf]

Supplementary Materials

Riccardo Vicedomini et al.

Contents

1 Supplementary Figures 1

2 Supplementary Tables 2

1 Supplementary Figures

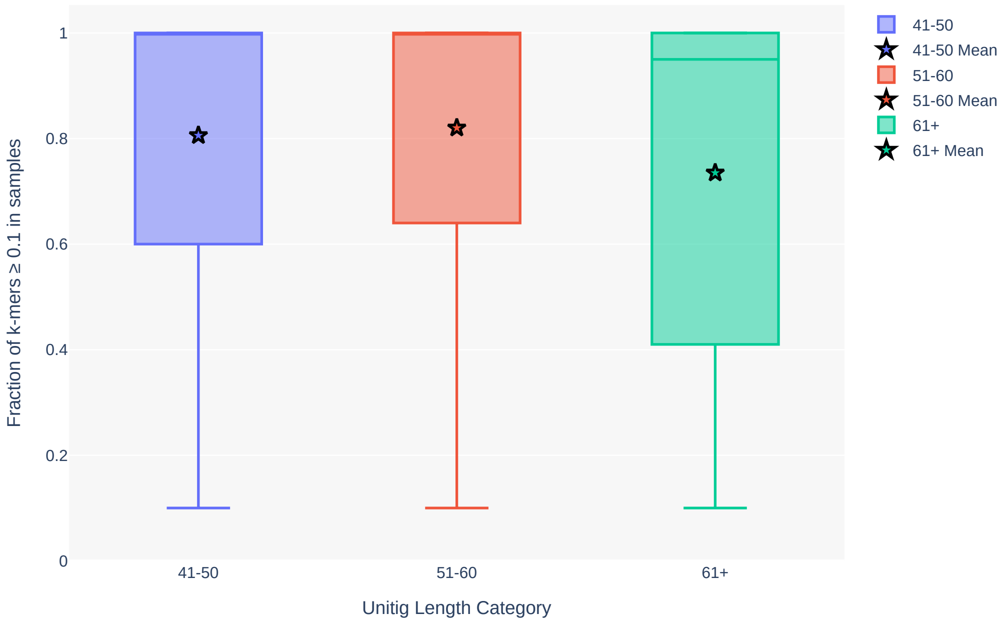

Figure 1: Boxplot of frequency of k-mers in a unitig per sample vs unitig length for small dataset. We filtered out frequencies that were ≤ 0.1

2 Supplementary Tables

Table 1: E. coli unitig statistics. Number of k-mers and unitigs across increasing number of *E. coli* assembled genomes extracted from AllTheBacteria

Number of samples	Distinct k-mers (M)	Unitigs (M)	Avg unitig length	Unitig/k-mer ratio
10	15.20	0.49	60.7	0.03
100	38.28	1.73	52.1	0.05
1000	82.33	4.24	49.4	0.05
2000	105.68	5.71	48.5	0.05
