# Supplementary figures and images for: MUSET: set of utilities for constructing abundance unitig matrices from sequencing data
Source: Bioinformatics. 2025 Feb 3;41(3):btaf054. doi: 10.1093/bioinformatics/btaf054 (PMC11897428; doi:10.1093/bioinformatics/btaf054)

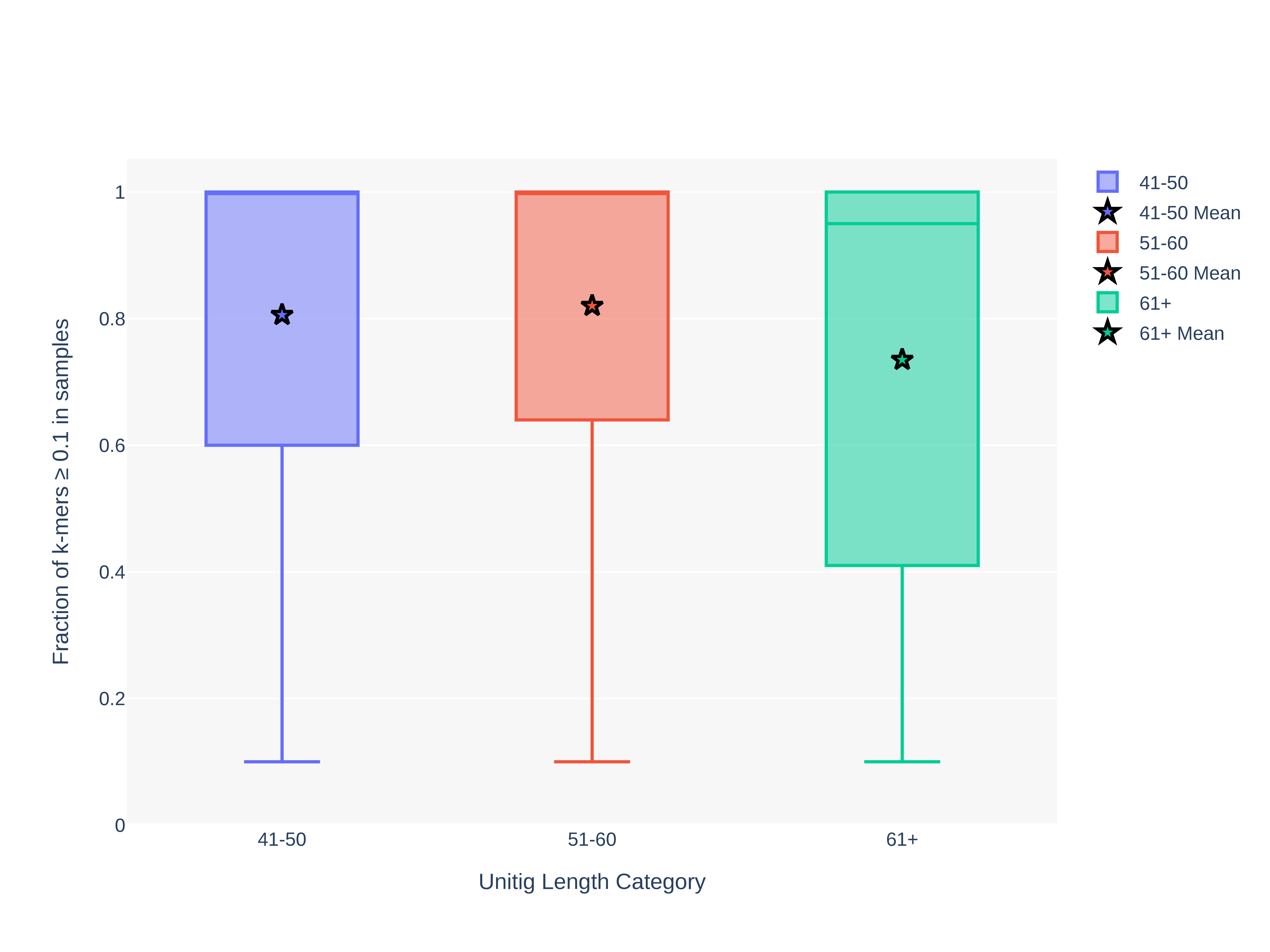

Supplement: btaf054_Supplementary_Data [file btaf054_supplementary_data.zip › Supplementary_Figure_S1_1200DPI.png]
